# A potential role for CCN2/CTGF in aggressive colorectal cancer

**DOI:** 10.1007/s12079-016-0347-5

**Published:** 2016-09-10

**Authors:** Inge Ubink, Elisha R. Verhaar, Onno Kranenburg, Roel Goldschmeding

**Affiliations:** 1 Cancer Center, University Medical Center Utrecht, Heidelberglaan 100, 3584CX Utrecht, The Netherlands; 2 Department of Pathology, University Medical Center Utrecht, Heidelberglaan 100, 3584CX Utrecht, The Netherlands; 3 University Medical Center Utrecht, PO Box 85500, 3508GA Utrecht, The Netherlands

**Keywords:** Colorectal cancer, Connective tissue growth factor, CCN2, Fibrosis

## Abstract

CCN2, also known as connective tissue growth factor (CTGF) is a transcriptional target of TGF-β signaling. Unlike its original name (“CTGF”) suggested, CCN2 is not an actual growth factor but a matricellular protein that plays an important role in fibrosis, inflammation and connective tissue remodeling in a variety of diseases, including cancer. In pancreatic ductal adenocarcinoma, CCN2 signaling induces stromal infiltration and facilitates a strong tumor-stromal interaction. In many types of cancer, CCN2 overexpression has been associated with poor outcome. CMS4 (Consensus Molecular Subtype 4) is a recently identified aggressive colorectal cancer subtype, that is characterized by up-regulation of genes involved in epithelial-to-mesenchymal transition, TGF-β signaling, angiogenesis, complement activation, and extracellular matrix remodeling. In addition, a high influx of stromal fibroblasts contributes to the mesenchymal-like gene expression profile of this subtype. Furthermore, compared with the other three CMS groups, CMS4 tumors have the worst prognosis. Based on these observations, we postulated that CCN2 might contribute to colorectal cancer progression, especially in the CMS4 subtype. This review discusses the available literature on the role of CCN2 in colorectal cancer, with a focus on the ‘fibrotic subtype’ CMS4.

## Introduction

Colorectal cancer (CRC) is one of the most common causes of cancer-related mortality in the Western world (Siegel et al. 2016). CRC is a highly heterogeneous disease. Traditional CRC staging takes into account the depth of invasive growth, the histological differentiation grade and the presence of metastases in lymph nodes and in distant organs. More recently, the CRC Subtyping Consortium has identified four Consensus Molecular Subtypes (CMS) based on integrative analysis of molecular data linked to tumor phenotype and clinical features (Guinney et al. 2015). CMS1 tumors are often microsatellite instable and are characterized by strong activation of immune pathways. This subtype responds best to immune checkpoint inhibition, presumably due to a deficient mismatch repair pathway, which leads to a high mutational load and the generation of tumor-specific neo-antigens (Le et al. 2015; de Weger et al. 2012). Tumors of CMS2 are characterized by a high level of copy number alterations, epithelial differentiation, up-regulation of *WNT* and *MYC* targets, and chromosomal instability. CMS3 tumors are likely to harbor activating mutations in *KRAS*, and show a marked deregulation of metabolic pathways; CMS3 tumors also display high-level chromosomal instability (CIN). CMS4 tumors are characterized by high expression of genes involved in epithelial-to-mesenchymal transition (EMT), Transforming Growth Factor β (TGF-β) signaling, angiogenesis and extracellular matrix remodeling. CMS4 tumors also exhibit a high density of tumor-associated fibroblasts. Importantly, CMS4 tumors have a poorer prognosis compared with the other subtypes, due to an increased likelihood of distant relapse (Guinney et al. 2015; Becht et al. 2016).

CCN2, is well-known for its involvement in connective tissue remodeling in various diseases, including cancer. CCN2 is a transcriptional target of TGF- β, that binds growth factors, extracellular matrix protein, and cell surface molecules, including integrins, thereby modulating signaling activity in multiple pathways (reviewed by (Lau 2016; Cicha and Goppelt-Struebe 2009)). In preclinical studies of pancreatic ductal adenocarcinoma (PDAC), a cancer type with particularly strong tumor-stroma interaction and stromal infiltration, CCN2 has been identified as a major determinant of tumor growth and metastasis, and as a valid therapeutic target (Dornhofer et al. 2006; Bennewith et al. 2009). A phase 1/2 trial with neo-adjuvant xanti-CCN2 (“anti-CTGF”) antibody treatment (FG3019) in locally advanced PDAC is underway (NCT02210559). Furthermore, CTGF overexpression has been associated with poor prognosis in several tumor types, including B-cell acute lymphoblastic leukemia (Boag et al. 2007; Sala-Torra et al. 2007), breast cancer (Chien et al. 2011), gastric cancer (Liu et al. 2008), glioma (Xie et al. 2004), melanoma (Hutchenreuther et al. 2015), pancreatic cancer (Bennewith et al. 2009) and prostate cancer (Yang et al. 2005). Based on the findings in other cancer types, and on the notion that CMS4 tumors are stroma-rich and have a poor prognosis, we suggest that CCN2 could also play a role in the fibrotic response that characterizes CMS4 CRC.

## CCN2 and colorectal cancer progression

TGF-β acts as a tumor suppressor in colorectal cancer initiation, by inhibiting excessive cell proliferation. However, in later stages of tumor development, TGF-β plays a crucial pro-metastatic role and its expression is associated with worse outcome (Massague 2008; Calon et al. 2012). mRNA and protein levels of CCN2, a well-known transcriptional target of TGF-β, were found to be increased in more advanced Duke’s and TNM stages of CRC (Ladwa et al. 2011).

CRC can metastasize via blood or lymphatic vessels. TGF-β signaling has been implicated in formation of metastases and facilitation of secondary organ colonization (Calon et al. 2012). By recruiting myofibroblasts to the invasive tumor front via TGF-β signaling, tumor cells facilitate their own invasion and metastatic spread (Massague 2008). A similar role has been proposed for CCN2 based on the observed correlation of CCN2 expression with α-smooth muscle actin expression in ileal carcinoids, an endocrine carcinoma that is extremely rich in fibrosis (Cunningham et al. 2010). Furthermore, conditioned medium from colon cancer cells can stimulate CCN2 expression in peritoneal fibroblasts (Yokota et al. 2016).

Although the contribution of lymphangiogenesis to CRC progression is currently unknown, it does correlate with poor prognosis (Sundlisaeter et al. 2007). We recently identified involvement of CCN2 not only in fibrotic matrix remodeling but also in lymphangiogenesis downstream of TGF-β in experimental kidney fibrosis (Kinashi et al., unpublished data). TGF-β activates CCN2 expression through canonical pSmad2/3 signaling, but depending on cell type and context also through RhoA, Ras, and MEKK 1/2 (reviewed by (Cicha and Goppelt-Struebe 2009)). The TGF-β pathway regulates lymphangiogenesis and stromal infiltration and activation, which may in part be mediated by induction of its transcriptional target CCN2.

## CCN2 and prognosis of colorectal cancer

**Fig. 1:**
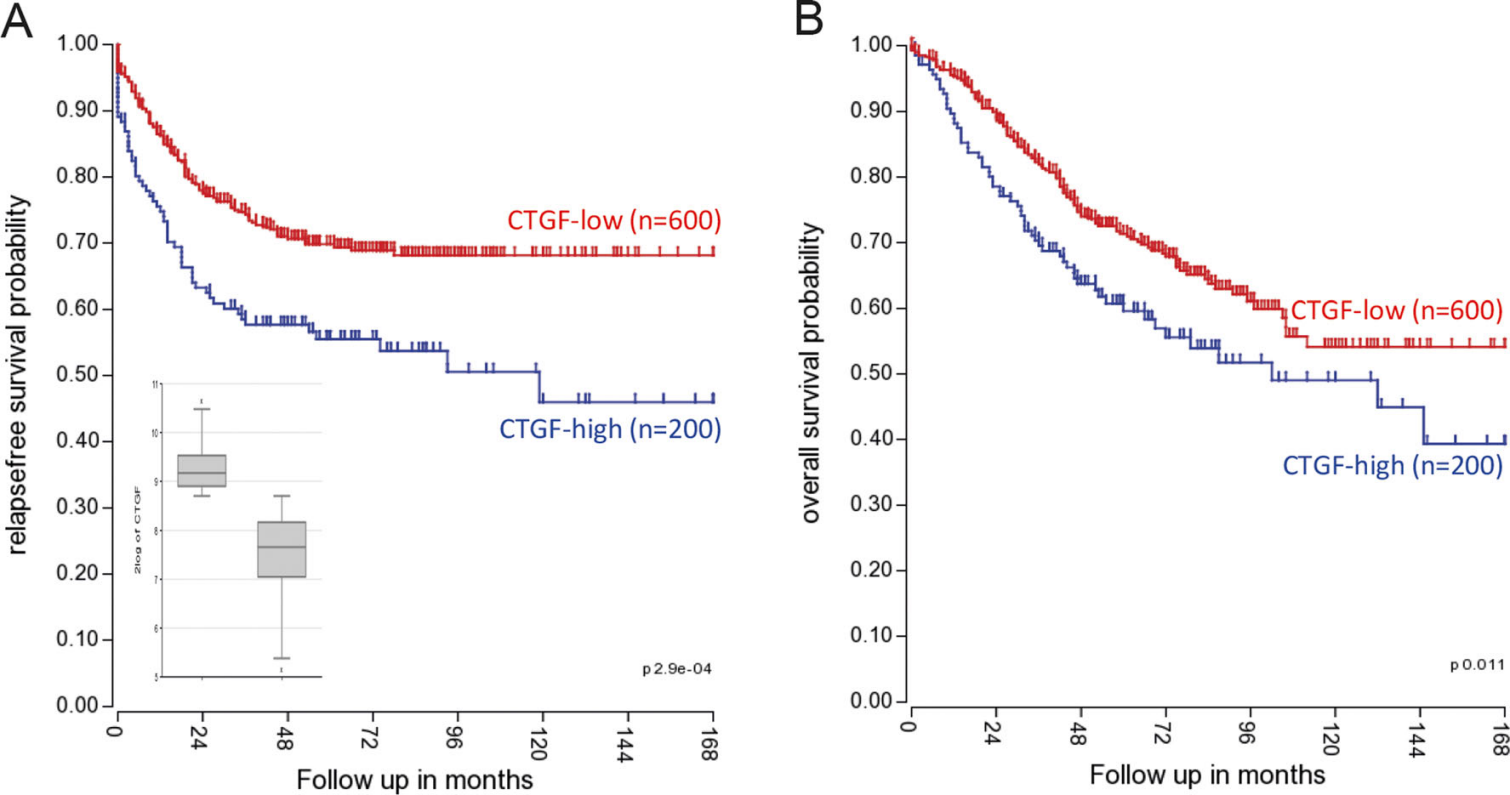
High CCN2(/CTGF) expression is associated with poor prognosis Kaplan Meier curves showing the disease-free (**a**) and overall (**b**) survival of the CCN2(/CTGF)-high (upper 25 %) and CCN2(/CTGF)-low (lower 75 %) expression subgroups. The inset shows the expression of CCN2(/CTGF) in the high and low subgroups

Several research groups have studied the relationship between CCN2 expression and CRC prognosis, with conflicting results. Lin and others (2005; 2011) performed immunohistochemical analyses of CCN2 expression in primary CRC, and found that high CCN2 levels were associated with a decreased risk of peritoneal metastasis and with significantly higher disease-free and overall survival. In other studies however, CCN2 levels were higher in more advanced CRC stages (Ladwa et al. 2011; Guo et al. 2015), and activation of the TAZ-AXL-CCN2 axis in CRC was associated with higher stage, higher grade and poorer survival in CRC patients (Yuen et al. 2013; Zhang et al. 2016). Using gene expression data from 800 primary CRC samples (from publically available data sets used by Guinney et al. (2015)), we found that relapse-free survival as well as overall survival of CRC patients with high CCN2 expression (*N* = 200; top-25 %) was significantly shorter than in CRC patients with low CCN2 expression (*N* = 600; lower 75 %), *p* < 0.001 and *p* = 0.011, respectively (Fig. 1a, b). Taken together, the data on CCN2 expression in relation to prognosis in CRC remains inconclusive. To resolve this issue, new studies are needed that couple the use of well-validated anti-CCN2 antibodies on immunohistochemistry with RNA analyses in thoroughly annotated large cohorts of CRC patients.

CCN2 expression has been linked to drug resistance in several types of cancer (Tsai et al. 2014; Lai et al. 2011; Yin et al. 2010). It has also been suggested that CCN2 expression contributes to therapy resistance in CRC, since overexpression of CCN2 induced resistance to radiotherapy (combined with simvastatin) in colorectal cancer cell lines, whereas CCN2 knockdown increased the radiosensitivity (Lim et al. 2015). Data on this subject are very limited however, and more research is needed to elucidate the role of CCN2 in the response to standard chemotherapy and targeted therapy in CRC.

## CCN2 and CMS4

**Fig. 2:**
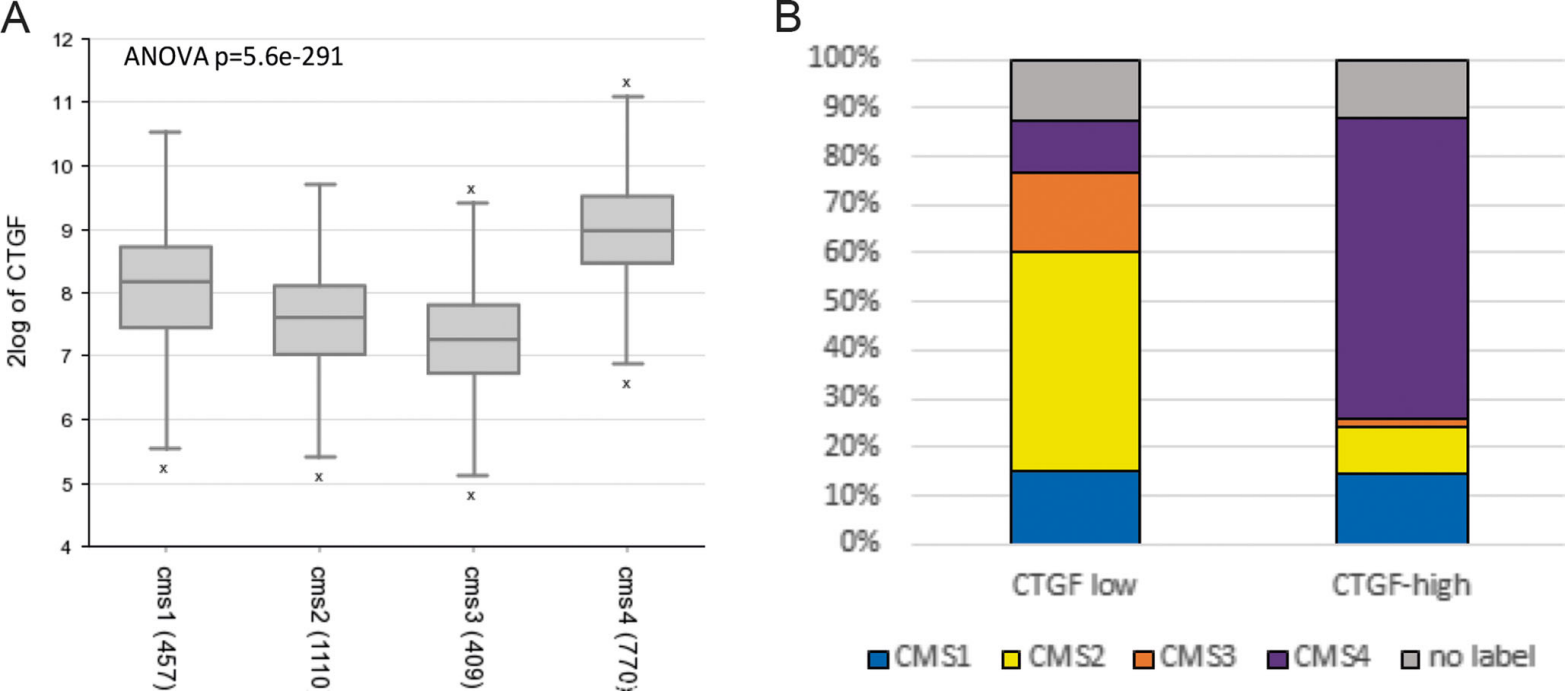
CCN2(/CTGF) and CMS (**a**) CCN2(/CTGF) mRNA expression in the consensus molecular CRC subtypes. (**b**) Analysis of the percentage CMS1–4 contributing to the CCN2(/CTGF)-high and CCN2(/CTGF)-low subgroups. The genomics analysis and visualization application R2 (http://r2.amc.nl) was used to analyze the expression of CCN-2(/CTGF) in the four consensus molecular subtypes (CMS1–4)

In a collection of 2476 primary CRC samples (Guinney et al. 2015), we compared the expression levels of CCN2(/CTGF) mRNA between the four CMS of CRC. This revealed that expression is particularly high in CMS4 (Fig. 2a). Furthermore, CMS4 tumors were strongly enriched in the CCN2(/CTGF)-high group, while the canonical subtypes (CMS2, CMS3) were enriched in the CCN2(/CTGF)-low subgroup (Fig. 2b). Interestingly, CCN2 was identified as an upstream activating regulator of a 19 gene-based risk profile that predicts poor CRC prognosis and benefit from adjuvant chemotherapy (Kim et al. 2014). Considering the strong association between CCN2 expression and the CMS4 colorectal cancer subtype, CCN2 could be an interesting target for therapy.

## Conclusion

The available data on the role of CCN2(/CTGF) in colorectal cancer progression and prognosis is limited, and in part contradictory. The high expression of CCN2(/CTGF)x in CMS4 colorectal cancer and its established role in tissue fibrosis warrant further investigation to establish the possible value of xCCN2(/CTGF) as a marker and potential therapeutic target in CRC, especially in the aggressive mesenchymal subtype.

## Abbreviations

CMS: Consensus Molecular Subtype
CRC: Colorectal cancer
CCN2: CCN-family protein 2
CTGF: Connective tissue growth factor
EMT: Epithelial-to-mesenchymal transition
PDAC: Pancreatic ductal adenocarcinoma
TGF-β: Transforming Growth Factor β
